# Development, Processing and Aging of Novel Zn-Ag-Cu Based Biodegradable Alloys

**DOI:** 10.3390/ma16083198

**Published:** 2023-04-18

**Authors:** Alexander Heiss, Venkat Sai Thatikonda, Andreas Richter, Lisa-Yvonn Schmitt, Daesung Park, Ulrich E. Klotz

**Affiliations:** 1 Department of Physical Metallurgy, Research Institute for Precious Metals and Metals Chemistry (fem), Katharinenstrasse 17, 73525 Schwaebisch Gmuend, Germany; 2 Department of Precision-Optics-Materials-Environment, University of Applied Sciences, 07745 Jena, Germany; 3 Physikalisch-Technische Bundesanstalt (PTB), 38116 Braunschweig, Germany; 4 Laboratory of Emerging Nanometrology (LENA), 38106 Braunschweig, Germany

**Keywords:** zinc alloys, biodegradable metal, precipitation hardening, thermomechanical treatment, mechanical properties, microstructure, aging

## Abstract

The use of biodegradable materials for implants is a promising strategy to overcome known long-term clinical complications related to permanent implants. Ideally, biodegradable implants support the damaged tissue for a certain period and then degrade, while the physiological function of the surrounding tissue is restored. Although Mg-based alloys nearly ideally lend themselves to biodegradable implants, a few critical shortcomings promoted the development of alternative alloy systems. Due to their reasonably good biocompatibility, moderate corrosion rate without hydrogen evolution and adequate mechanical properties, increasing attention has been paid to Zn alloys. In this work, precipitation-hardening alloys in the system Zn-Ag-Cu were developed relying on thermodynamic calculations. After casting the alloys, their microstructures were refined by thermomechanical treatment. The processing was tracked and directed, respectively, by routine investigations of the microstructure, associated with hardness assessments. Although microstructure refinement increased the hardness, the material proved to be susceptible to aging as the homologous temperature of zinc is at 0.43 T_m_. Besides mechanical performance and corrosion rate, long-term mechanical stability is another crucial factor that must be taken into consideration to ensure the safety of the implant and thus requires a profound understanding of the aging process.

## 1. Introduction

In the beginning, when biodegradable materials for orthopedic and vascular implants were conceptualized, scientific research was mainly focused on the polymers poly (glycolic acid) (PGA), poly (L-lactic acid) (PLLA) or poly (lactic acid-co-glycolic acid) (PLGA). However, due to the inherent disadvantageous properties of these polymeric materials such as poor mechanical properties, inhomogeneous dissolution involving the release of acids, the potential clinical use cases seem to be rather limited. Meanwhile, magnesium-based alloys gained momentum, most notably due to their excellent biocompatibility. However, despite intense R&D on Mg-based alloys for over a decade and substantial financial backing, a broad commercial breakthrough has not yet been achieved.

The low electrode potential of Mg (−2372 V) leads to a relatively high corrosion rate [1]. In combination with some other undesirable characteristics of Mg corrosion, such as intergranular corrosion, pitting corrosion, or stress corrosion cracking, this may lead to premature implant failure [2]. In addition, the corrosion of Mg-alloys involves the formation of hydrogen gas. As Mg corrosion is related to its inherent properties, they can hardly be mitigated by alloying or additional processing. For example, alloying with Zn has multiple effects. Zn is known to act as a grain refiner. Furthermore, Zn has a high solubility in Mg which leads to solid solution strengthening and enables precipitation aging. Thus, alloying with Zn improves mechanical properties. On the other hand, a lower potential difference between the phases has a negative effect on the corrosion rate [3]. Other advanced approaches to improve the corrosion resistance are available, including the application of a coating or the creation of an oxide layer [2,4]. As the problematic aspects of Mg corrosion remain unresolved, two new potential biodegradable metals, Zn (−0.762 V) and Fe (−0.440 V), have attracted growing scientific interest. While the excellent mechanical properties of Fe are appealing, some studies concluded that the biocorrosion rate of Fe is too low so the development of additional counterstrategies is required [5]. Early in vivo studies of pure Zn as biodegradable material for stents demonstrated that the metal exhibited a steady corrosion rate and did not increase inflammation, thrombosis, or restenosis [6,7]. The authors also pointed out that an elaborate alloy development is required to tune the properties as pure Zn shows insufficient mechanical properties for implant applications. Other studies proved good biocompatibility, good hemocompatibility, and a promising healing process of Zn-based alloys [8,9].

Based on toxicity considerations, several alloying elements have been evaluated, such as Li [10,11,12,13,14], alkaline earth metals (Mg, Ca, Sr), Fe [15], Cu, precious metals (Ag [16,17,18], Au [19]) and rare earth elements (Er, Dy, Ho) [20]. Furthermore, grain refiners, such as V [19], Mn [10,21], or Zr [16] have been added. Among those elements, Mg, Li, Mn, Ag and Cu were identified as the most promising ones regarding their secondary phases and the resulting impact on microstructure, strength, ductility, corrosion rate, and cytotoxicity [22]. Recent studies indicate that alloying Zn with Ag and Cu, respectively, represents a promising step towards meeting all the requirements for biodegradable metals [17,18,21,23]. Alloy composition and the thermomechanical treatment (TMT) affect the Zn matrix as well as the volume fraction and the morphology of the secondary ε-phase. In contrast to Cu, Ag generally improves the strain-hardening effect [18]. Nobler elements tend to increase the corrosion rate due to microgalvanic elements. However, since the TMT history affects the balance between the amount of solved alloying elements in the anodic Zn matrix versus the volume fraction, size, and distribution of the cathodic, secondary phase particles, the effective corrosion potential may vary. Therefore, some studies on Zn alloys with nobler elements reported either an increased corrosion rate [17,18] or a rather decreased corrosion rate [19,21].

With regard to vascular applications, Zn0.1Li exhibits good biocompatibility, good mechanical properties (UTS 274 MPa, 17%), an adequate biocorrosion rate and a low inflammatory response [11]. Zn0.8Li in combination with optimized processing resulted in a UTS of 365 MPa and an elongation of 22% which are significantly above the recommended benchmarks, UTS > 300 MPa, >15% elongation, for implants [13,24,25]. The alloying element Mn has good biocompatibility and functions as a grain refiner [21,26]. Zn0.8Li0.8Mn is characterized by an UTS ~510 MPa and an exceptional elongation of 103% [27]. The authors concluded that such Zn-based alloys have the potential to close the gap between biodegradable and conventional implants.

As some researchers already have pointed out, focusing on alloy optimization in order to meet the required mechanical benchmarks and corrosion rates does not do justice to the complexity of biodegradable implants. Due to the relatively high homologous temperature of Zn (0.43 × T_m_), the effect of high-temperature deformation and diffusion-related processes should be considered [28,29]. Load-bearing orthopedic implants are prone to creep at body temperature [27]. Moreover, with regard to vascular implants, cases of low strain hardening rates [30] and even strain softening [21,31] have been reported. A weak strain hardening would promote the recoil of the stent. As strain softening is associated with limited uniform deformation, corresponding devices would be prone to unexpected failure.

Here, promising alloy variants in the system Zn-Ag-Cu system were identified based on thermodynamic simulations. The alloys were cast and then subjected to a systematic TMT, each step of the process was checked by structural investigations and hardness measurements. Given a homologous temperature of 0.43 T_m_, long-term aging effects were investigated as well. This pilot study suggested that aging effects can become relevant and thus need to be factored in regarding potential use cases as biodegradable implant material.

## 2. Materials and Methods

### 2.1. Thermodynamic Calculations

The phase diagrams were calculated using the Thermo-Calc software package version 2021a–2022b (Solna, Sweden) with the thermodynamic databases of TCMG6 and TCCU2.

### 2.2. Casting and Thermomechanical Treatment

600 g of each alloy was produced from high-purity elements (>99.9%) by induction melting (Indutherm VC 500 D; Walzbachtal/Woessingen, Germany) and gravity casting under 0.8 bar Argon in a graphite crucible. To that end, the crucible was pre-heated to about 200 °C before filling with Zn, Ag, and Cu. Regarding the quaternary Li alloys, either pure Li or an Ag-6Li master alloy, was added first and then covered with the remaining material. Likewise for Mn, a Cu-30Mn master alloy was added. The material was melted at 650 °C, induction-stirred for 10 min and then cast into a graphite mold. Moreover, 12 kg of Zn-2.5Ag-1.5Cu-0.1Ti were cast under Ar gas flow at Meotec GmbH (Aachen, Germany). The alloys were subjected to two annealing—deformation iterations. Beforehand, a pilot study to assess the optimal annealing condition was conducted by varying temperatures (350–400 °C) and time (6–8 h). In agreement with previous reports, a higher temperature and a longer heating period proved to be beneficial. Thus, the as-cast (AC) alloy was homogenized, i.e., solution annealed, at 400 °C for 8 h [21,32] under argon atmosphere (Nabertherm furnace, Lilienthal, Germany) and quenched in water. The composition of all alloys was assessed by inductively-coupled plasma optical emission spectrometry (ICP-OES) analysis and X-ray fluorescence analysis (XRF), respectively (Table A1). Next, the alloys were 50% hot rolled at 200 °C, followed by another annealing step at 400 °C for 30 min and water quenching. Finally, the alloys were 50–75% cold rolled.

### 2.3. Microstructure Characterization

Metallographic cross-sections of each processing step were prepared and investigated by optical microscopy (OM, Axioplan 2; Carl Zeiss Microscopy GmbH, Jena, Germany) [17]. The grain size was assessed according to the ASTM E112 based on light microscopic images of metallographic cross sections using ImageJ/FIJI software 1.53f-t [33]. As rolled microstructures were excluded as they were characterized by flattened, high axis-ratio grains with blurred boundaries (OM). The volume fraction of the secondary phase was quantified using the trainable Weka segmentation plugin included in the ImageJ/FIJI [34].

The Vickers hardness HV1 was assessed in triplicates on metallographic cross-sections (KB 30BVZ, KB Prüftechnik GmbH, Hochdorf-Assenheim, Germany). Cold-rolled sheets (CR) were subjected to an X-ray diffraction based phase analysis as described previously [17]. Furthermore, the microstructure was investigated by field-emission scanning electron microscopy (FE-SEM, Zeiss Auriga 60, Oberkochen, Germany) on metallographiccross-sectionss. To this end, the cross sections were polished by broad Ar ion beam milling (Hitachi IM 4000, Chiyoda, Japan) at 6 kV and 6° for 10 min. Images were acquired at an acceleration voltage of 15 kV with a 60 µm aperture using the secondary electron detector (SE) and backscattered electron detector (BSE), respectively. Local element compositions were analyzed at 15 kV with a 30 µm aperture by energy dispersive X-ray spectrometry (EDX, X-Max 80, Oxford Instruments, Abingdon, UK).

For a detailed nanostructure investigation, a cross-sectional specimen of the aged Zn-2.5Ag-1.5Cu-0.15Li alloy was prepared by using a dual focused ion beam (FIB) (Thermo Fisher Helios 5UX, Waltham, MA, USA). In order to minimize the beam damage and undesirable intermetallic phase formation resulting from the Ga beam ions, low kV milling at 2 kV was performed at the final preparation step [35]. The cross-sectional specimen was investigated by a double aberration-corrected transmission electron microscope (TEM, JEOL NeoARM 200F, Akishima, Japan) equipped with a cold field emission gun (FEG) and electron energy loss (EEL) spectrometer (Gatan Enfinium, Pleasanton, CA, USA).

### 2.4. Thermal Analysis

Differential Thermal Analysis (DTA) experiments were conducted (Netzsch STA 449C, Selb, Germany). The temperature calibration of the equipment was performed with pure indium, silver, gold, and nickel. For each sample, two continuous heating and cooling curves were acquired at a rate of 10 K/min. The first cycle started with heating from 25 °C to 550 °C, followed by cooling to 200 °C. In the next cycle, the sample was heated again to 550 °C, followed by cooling to 25 °C.

### 2.5. X-ray Diffraction

Phase analysis of bulk samples was performed by X-ray diffraction in Bragg-Brentano-Geometry using a diffractometer D8 discover Da Vinci (Bruker AXS GmbH, Karlsruhe, Germany) equipped with a 1D Lynxeye-XET (Bruker AXS GmbH, Karlsruhe, Germany) detector using copper radiation. A fixed divergence slit of 0.3° was employed to keep the radiated volume of the sample constant during the measurement.

For the phase analysis on cross sections, a setup with spatial resolution in diffractometer D8 discover (Bruker AXS GmbH, Karlsruhe, Germany) was used. The X-ray beam of copper Kα was focused on the sample by primary beam optics (Goebel mirror, pinhole collimator 1 mm, and snout 1 mm), and the diffracted beam was collected using an energy discriminating Lynxeye-XET detector in 1D mode. It is noted that some texture effects may be related to the limitation in spot size.

Phase was analyzed by comparing measured reflections with the ICDD-PDF2 database (International Centre for Diffraction Data—Powder Diffraction File) and to the COD database (Crystallography Open Database). Additional Rietveld analyses were performed using Topas v5 (Bruker AXS GmbH, Karlsruhe, Germany) for the evaluation of phase content and the lattice parameter determination. Some minor reflection peaks in the pattern were attributed to the formation of further intermetallic phases in minor concentration due to local inhomogeneity.

## 3. Results

### 3.1. Alloy Development

**Figure 1:**
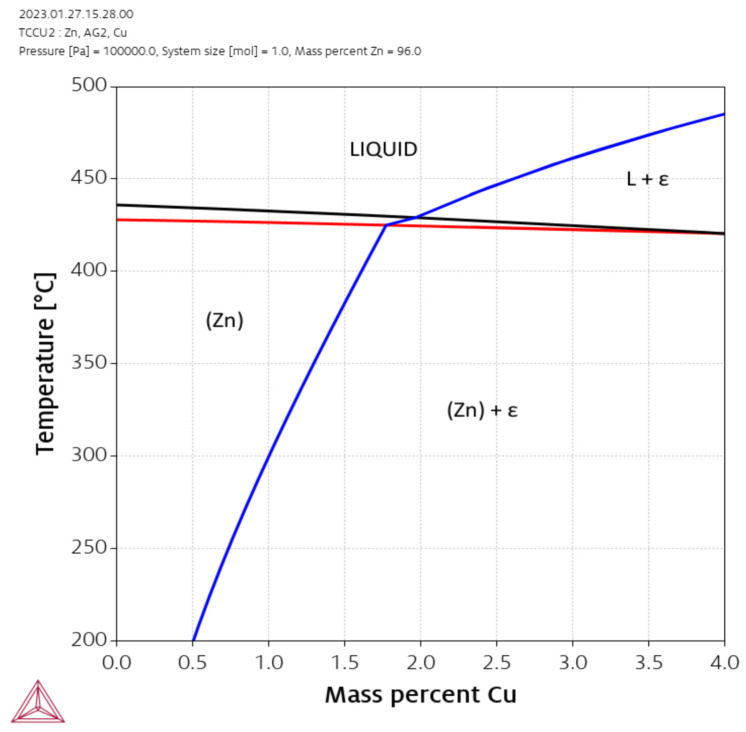
Isopleth section of the Zn-Ag-Cu system at 96 % zinc.

Novel biodegradable alloys were developed based on the predictions from thermodynamic calculations using the ThermoCalc software package. Based on our previous experiments [17,19] and on information from the literature [21,32] a zinc content of 96% was chosen, which leads to the formation of a stable Zn solid solution [36]. The solid solution containing Cu, Ag, and Zn forms the Hume-Rothery ε-phase with a hexagonal close-packed (hcp) structure in a very wide composition range. Figure 1 shows a calculated isopleth section of the Zn-Ag-Cu system at 96% zinc with the ε-phase and the zinc solid solution phase (HCP_A3). Two compositions in this section were targeted: an Ag-rich composition (2.5 wt% Ag, 1.5 wt% Cu) and a Cu-rich composition (1.5 wt% Ag, 2.5 wt% Cu). The simulation predicted that the ε-phase in the Ag-rich alloy should dissolve completely during annealing while the Cu-rich alloy is predicted to have the ε-phase already precipitated from the melt. The later alloy should therefore be more stable against grain coarsening during annealing. In agreement with another study however, it was found experimentally that the ε-phase in the as cast microstructure (AC) could only be removed completely in Zn-1.5Ag-1.5Cu by solution annealing [32]. The ε-phase could not be dissolved in any other alloy with higher Ag or Cu content.

**Figure 2:**
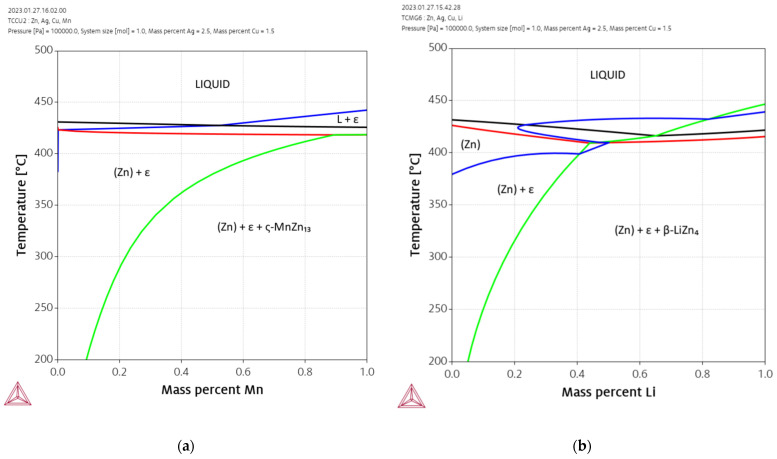
Effect of the additional alloying elements Mn (**a**) and Li (**b**) on the base alloy Zn-2.5Ag-1.5Cu.

The effect of additional alloying elements Mn and Li on the Zn-Ag-Cu system is shown in Figure 2. In these calculations, Zn was substituted by Mn and Li, respectively, while the contents of Ag and Cu were kept constant at 2.5% and 1.5%, respectively. The binary Mn-Zn is very complex due to the numerous intermetallic phases and the four allotropic modifications of Mn. Mn is a strong stabilizer of the ε-phase. Hence, the addition of extremely small amounts of Mn results in the formation of an ε-phase which is then stable up to the solidus temperature. The binary intermetallic phases with the highest Zn content are ζ-MnZn_13_ and δ-MnZn_9_ [37]. However, ζ-MnZn_13_ is not included in the thermodynamic database. Therefore, MnZn_9_ is the intermetallic compound with the highest Zn content that is stabilized by larger additions of Mn. The addition of Li also increases the stability of the ε-phase, but the effect is weaker compared to Mn. Only a small window above 400 °C exists allowing the complete solution annealing of the alloy. The simulation indicates that the phase LiZn_4_ is formed in significant amounts at contents above ca. 0.15% Li.

**Figure 3:**
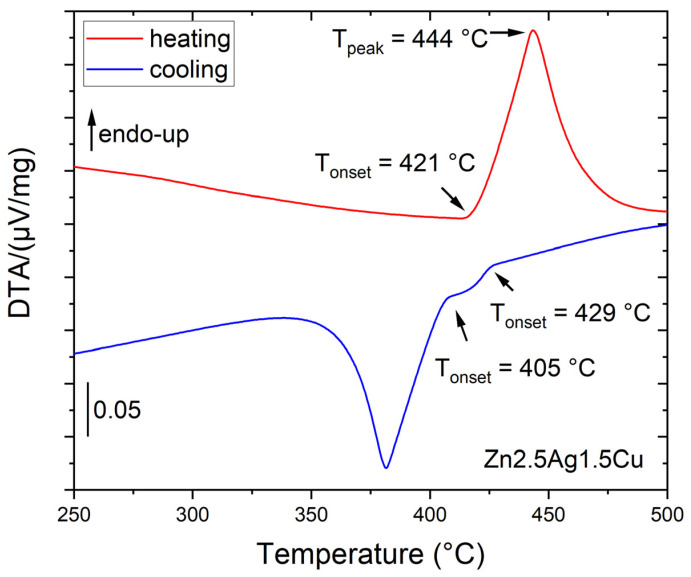
DTA of Zn-2.5Ag-1.5Cu.

Three Zn-2.5Ag-1.5Cu-(Mn|Li) alloys were investigated by differential thermal analysis (DTA), i.e., subjected to continuous heating and cooling measurements at a rate of 10 K/min. As an example, Figure 3 shows the course of the experiment for Zn-2.5Ag-1.5Cu. The onset of the endothermic peak observed at 421 °C reflects the onset of melting. The peak temperature at about 444 °C reflects the end of melting. The cooling curves suggest a primary crystallization at about 429 °C and a subsequent peritectic transformation at about 405 °C. Table 1 confirms that the values obtained by simulation and by DTA are in reasonable agreement.

The ε-phase is the primary phase formed from the melt. The primary dendrites are partially reacting with the melt to form Zn: L + ε => Zn. The peritectic reaction, which is nonvariant in the binary systems, becomes monovariant in the ternary system. This monovariant line connects the peritectic points of the binary systems [38].

### 3.2. Alloy Processing

**Figure 4:**
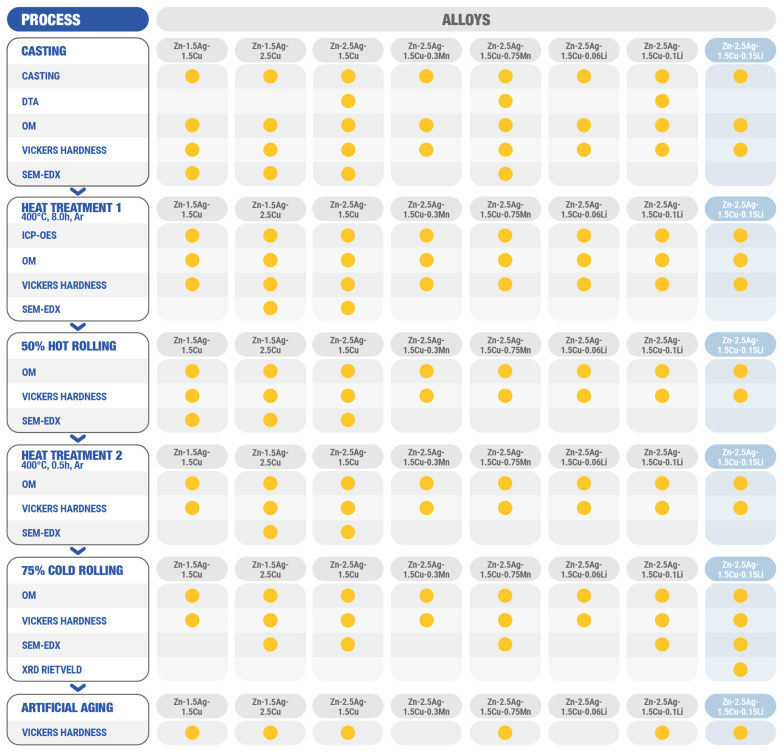
Schematic overview of all alloys, processing steps, and methods. After casting, the microstructure was refined by TMT, followed by an aging study of the cold-rolled principal alloys. Zn-2.5Ag-1.5Cu-0.15Li (bluish right column) was studied in more detail involving another heat treatment. DTA: differential thermal analysis, OM: optical microscopy, SEM: scanning electron microscopy, EDX: energy dispersive X-ray spectroscopy, XRD: X-ray diffraction, Rietveld analysis.

Plastic deformation and heat treatment cycles, subsumed hereinafter under the term thermomechanical treatment (TMT), were employed to improve the mechanical properties of Zn-alloys by microstructure refinement [12,14,21,32,39]. For the sake of clarity, all alloys as well as their processing pathways are compiled in Figure 4.

### 3.3. As Cast Alloys

**Figure 5:**
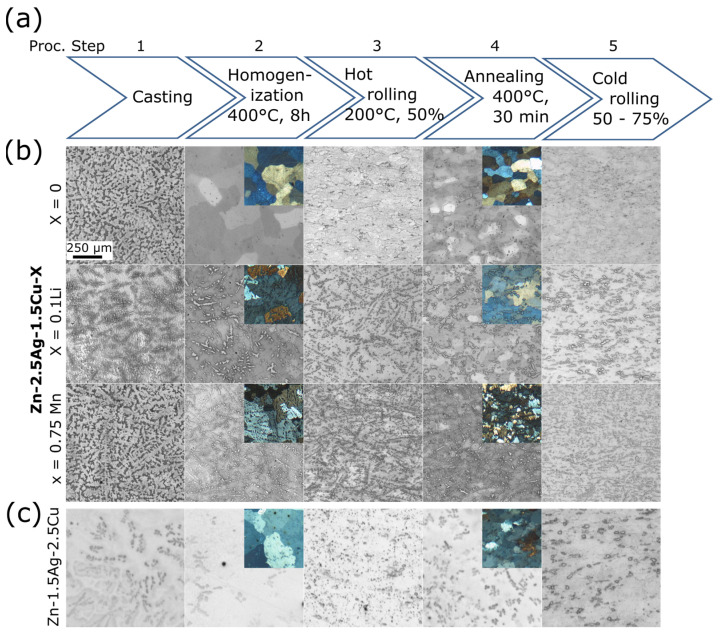
OM investigation of the TMT-related refinement of the microstructure. (**a**) Numbered processing steps for microstructure refinement. (**b**) microstructure of three Zn-2.5Ag-1.5Cu-X alloy variations. The insets show corresponding polarized light micrographs at the same scale. (**c**) Alloy with inverted Ag/Cu ratio.

**Figure 6:**
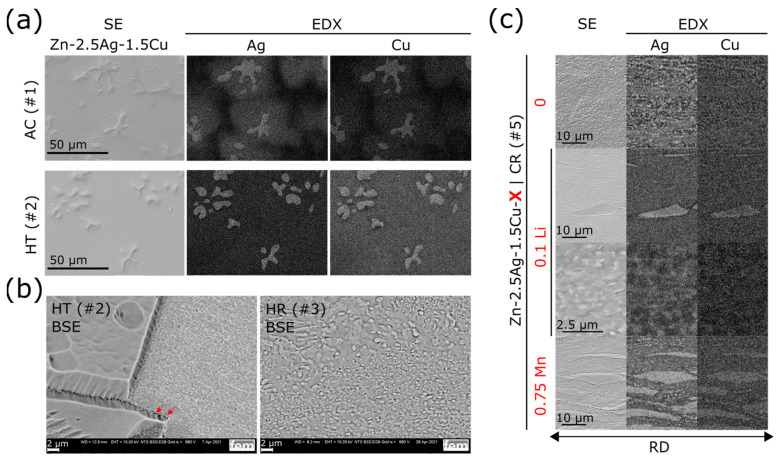
SEM investigation of the effect of TMT on the ε-phase transformation. Processing step numbers were assigned according to the scheme in Figure 5a. (**a**) AC Zn-2.5Ag-1.5Cu cross section depicting the dendritic ε- phase embedded in the η-Zn matrix. Segregations around the secondary phase were observed. Heat treatment (HT, i.e., homogenization) caused a partial dissolution of the secondary phase. Accordingly, the Zn solid solution showed an increased content of solutes. (**b**) (**left**): matrix grain boundaries after HT. The observed topography was due to ion polishing (see methods) as the ablation rate depends on the crystal orientation. Besides the dendritic phase, a few submicron ε-phase particles were found along the grain boundaries (red arrows). (**right**): Hot rolling (HR) induced dynamic precipitation. (**c**) Microstructure after cold rolling (CR) investigated by SE imaging and EDX elemental mapping. The morphology of the ε-phase is determined by elongated particles, i.e., flattened dendrites, and fine precipitates.

Analogous to Figure 4 and Figure 5a illustrates the sequence of all TMT steps. Figure 5b,c show the corresponding micrographs. The investigations by optical microscopy (OM) as well as by scanning electron microscopy (SEM) of the as-cast state (AC) revealed that a dentritic ε-phase embedded in the η-Zn matrix has formed (Figure 5b,c and Figure 6a). Solidification was associated with a peritectic reaction, i.e., the initial ε-phase partially dissolved and reacted with the liquid to form the Zn solid solution. This caused pronounced segregations around the dendritic ε-phase. After casting, the ingots were homogenized and quenched in water. All alloy compositions were then analyzed quantitatively by ICP-OES (Table A1). Only homogenization of Zn-1.5Ag-1.5Cu resulted in a complete dissolution of the dendritic secondary phase, while it was still present in all other alloys. The SEM-EDX analysis of homogenized Zn-2.5Ag-1.5Cu in Figure 6a shows the partial dissolution of the ε-phase and the resulting accumulation of solutes throughout the Zn matrix. On the other hand, the segregations stemming from the peritectic dissolution of the ε-phase were no longer detectable. Moreover, a few submicron sized ε-phase particles were identified along the boundaries of the large Zn matrix grains, (red arrows in Figure 6b, left).

### 3.4. Microstructure Refinement by Thermomechanical Treatment

The OM images in Figure 5b,c show the microstructure evolution of the principal alloys along the processing pathway. All quantitative data acquired during TMT is compiled in Table A2 (Appendix A). In comparison to the base alloy Zn2.5Ag1.5Cu, the addition of Li (0.1 wt%) had no significant effect on the nominal matrix grain size. However, the volume fraction of the secondary phase was noticeably increased. This caused a rather angular appearance of the Zn matrix grains (Figure 5b). Zn2.5Ag1.5Cu0.1Li proved to have the highest Vickers throughout all processing steps. As predicted by the simulation, alloying with Mn increased the volume fraction of the secondary phase even more. In addition, the well-known Mn-related grain refinement effect was observed [21,40]. Accordingly, the hardness of the Mn-containing quaternary alloy was considerably higher, compared to the base alloy.

AC Zn-1.5Ag-2.5Cu, i.e., with an inverted Ag/Cu ratio, showed a high-volume fraction of the dendritic ε-phase as well (Figure 5c). This is due to the lower solubility of Cu in Zn, compared to Ag. Subsequent TMT resulted in a decrease in the volume fraction of the secondary phase. However, the final cold rolling led to a slight resurgence of the secondary phase fraction.

In general, hot and cold rolling procedures (HR, CR) increasingly disrupted the dendrites, resulting in an elongated particle shape (Figure 5b,c and Figure 6c). Furthermore, rolling induced dynamic precipitation, i.e., precipitate formation of various sizes and non-uniform shapes (Figure 6b,c). The elemental maps obtained from the EDX analysis illustrate the distribution of the secondary phase particles after CR (Figure 6c). Compared to Mn, the Li addition induced the formation of considerably finer precipitates.

### 3.5. Phase Analysis

**Figure 7:**
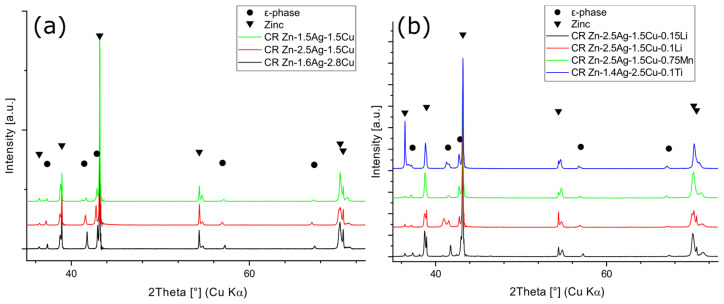
XRD phase analysis of CR alloys. Slight shifts in the Zn-phase reflection peak positions were observed, possibly reflecting small variations in the composition of the solid solution or staking faults. Furthermore, the XRD patterns confirm the presence of an ε-phase (Zn-Ag/Cu). (**a**) XRD patterns of ternary alloys, (**b**) XRD patterns of quaternary alloys.

Microstructure refinement and work hardening by creating and pinning dislocations, dendrite disruption, and precipitate formation improved the mechanical properties, indicated by an increase in Vickers hardness (Table A2). After TMT, the CR alloys were investigated by X-ray diffraction (XRD). The XRD analysis confirmed previous findings by SEM-EDX that all alloys consist of a Zn-matrix and of an embedded ε-phase (Figure 6c). Zn is considered here as one solid solution phase. However, slight shifts of the reflection peaks were noted (Figure 7a,b), suggesting local variations in chemical composition.

The Rietveld analysis of the XRD patterns revealed a diverging Zn c-parameter, while the Zn a-parameter was unaffected (Table 2). In contrast, the lattice parameters of the ε-phase were unaffected. The Li (≤0.15%) and Mn (≤0.75%) containing phases were not detectable by XRD due to the sensitivity limit. LiZn_4_ could only be detected in higher concentrated Zn-0.8Li by XRD [12]. Likewise, while no Mn-phases were detected in Zn-4Ag-0.6Mn, which is in agreement with our results, Zn-0.8Mn contained the phase ζ-MnZn_13_ [21,27].

### 3.6. Aging Behavior

**Figure 8:**
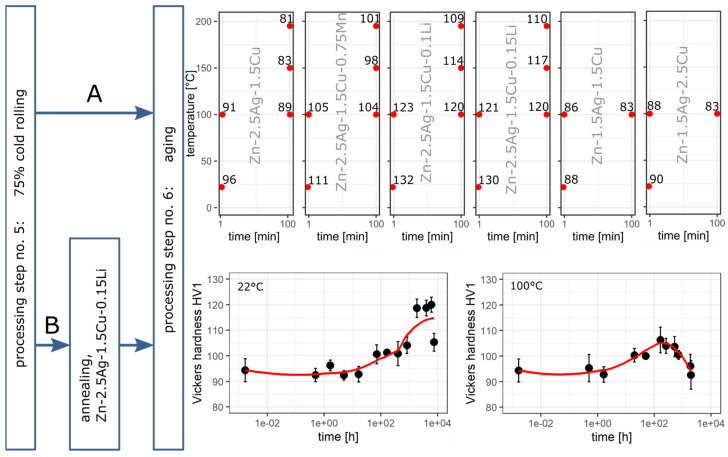
Investigation of aging by tracking the Vickers hardness. Path A: Effect of thermal treatment on the hardness of 75 % cold rolled alloys. Each red dot represents a t-T point with the corresponding hardness HV1. CR-related dynamic precipitation already maxed the precipitation potential out. Instead, recovery and coarsening effects became dominant leading to a decline in hardness. Path B: another heat treatment (400 °C, 30 min, Ar atmosphere) was prepended. Aging experiments were conducted at 22 °C and 100 °C over a period of up to 322 days.

At first, CR alloys were directly subjected to an artificial aging study. Path A in Figure 8 demonstrates that the thermal treatment resulted in an instant decrease in Vickers hardness in a time and temperature-dependent manner. Hence, CR-related dynamic precipitation already maxed the precipitation potential out, which is why the heat treatment only induced coarsening effects. Therefore, Path B included an additional heat treatment (400 °C, 30 min), giving rise to recrystallization, grain growth, and dissolution of precipitates. Zn-2.5Ag-1.5Cu-0.15Li was selected for this aging study because the Li-containing alloys tended to show the highest hardness levels. HT of the CR state resulted in a drop in hardness from 130 HV1 to 94 HV1. The subsequent aging experiment was conducted at 22 °C and 100 °C. As a control, the alloys were also stored at -18 °C for 10 months, resulting in only a slight increase in hardness to 99 HV1. At room temperature, the hardness started to increase after a lag period until the maximum hardness of 120 HV1 was reached within about 257 days. Following Arrhenius kinetics, after only 35 days at 100 °C the maximum hardness of 106 HV1 was reached, followed by a decline in hardness.

**Figure 9:**
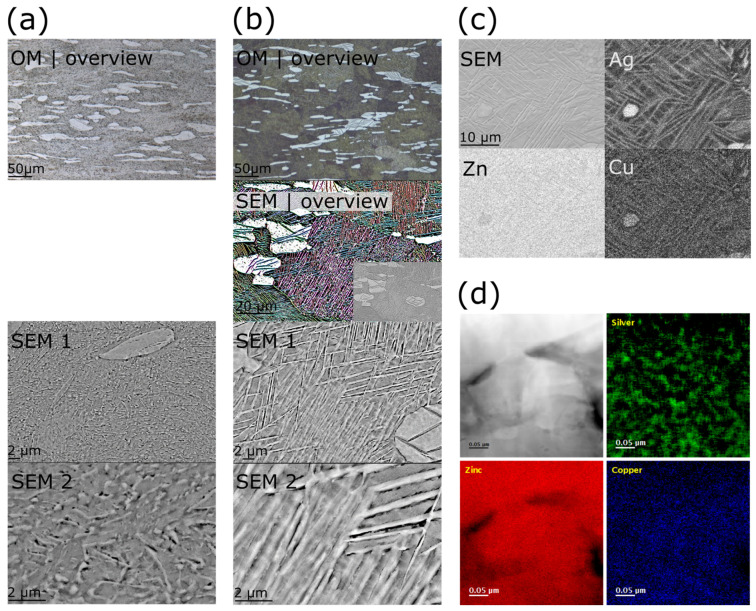
Investigation of overaged Zn-2.5Ag-1.5Cu-0.15Li by OM and SEM. (**a**) Aging at room temperature for 322 days, (**b**) aging at 100 °C for 83 days. The structures in the SEM-overview were colored according to their respective orientation. The original SEM image is shown in the inset. (**c**) EDX mapping including an SEM image of the artificially aged microstructure and the corresponding EDX element distributions. (**d**) STEM-EELS investigation of the needle-like structure of Zn-2.5Ag-1.5Cu-0.15Li after aging at 100 °C. While Zn and Cu tend to be homogeneously distributed, segregation results in an Ag pattern akin to a spinodal decomposition with structures in the range of 10–30 nm.

The micrographs in Figure 9 show the overaged microstructure of after natural aging for 322 days and artificial aging at 100 °C for 83 days, respectively. The micrographs in column (a) of Figure 9 show the microstructure after natural aging at increasing magnifications, while column (b) shows the corresponding micrographs after artificial aging. The image (b)-SEM-overview was colored according to the orientation of the structure to facilitate the identification of the intra-grain needles as well as of the grain morphologies. Figure 6b shows the microstructure typically observed after annealing. Starting from the annealed state, natural aging gave rise to the formation of fine precipitates associated with an increase in hardness (Figure 8b). The overaged microstructure contained predominantly elongated precipitates (Figure 9a). Artificial aging at 100 °C resulted in a dense network of interlocked needles (Figure 9b). In addition, instead of having a compact morphology, the coarse particles of the ε-phase were rather fragmented. The EDX elemental maps indicated that the needles were enriched in Ag and Cu (Figure 9c).

For a more detailed characterization of the formed needle-like microstructure inside the grains, the EEL spectrum imaging technique was employed. Figure 9d shows the EELS elemental maps for Zn-2.5Ag-1.5Cu-0.15Li aged at 100 °C. While the Ag map showed strong evidence for an inhomogeneous distribution of Ag in the matrix area, arguably homogeneous distributions of Zn and Cu were observed. In addition, the distribution pattern of Ag suggests a spinodal decomposition in a miscibility gap in the ternary phase field. The thickness of the Ag segregation patterns ranged from 10 to 30 nm. In our cautious EELS analysis, no Li-containing phase was detected.

**Figure 10:**
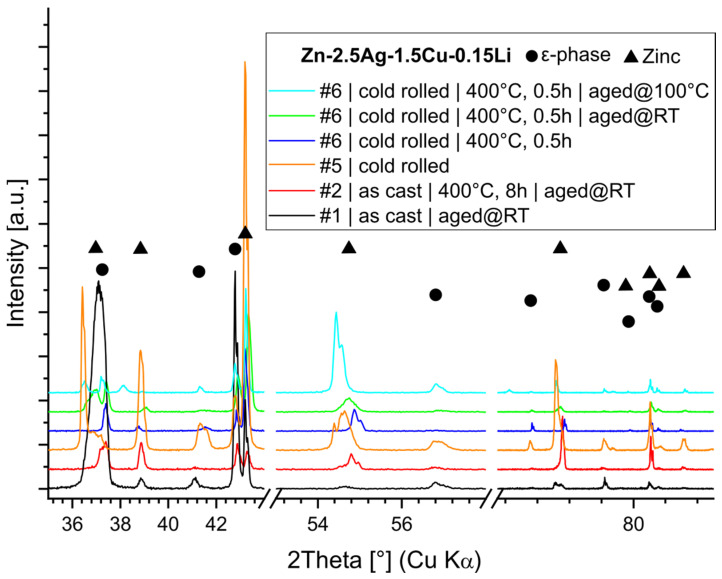
XRD analysis of Zn-2.5Ag-1.5Cu-0.15Li in various processing and aging states. The “aged @ 100 °C” pattern contains a few weak Ag reflections which are probably related to sample preparation.

Furthermore, the microstructure evolution of Zn-2.5Ag-1.5Cu-0.15Li, i.e., by TMT and aging, was tracked by XRD (Figure 10). Table 3 contains the results of the Rietveld analysis along with the mechanical properties. The XRD analysis revealed that CR resulted in a pronounced increase in the c-parameter of the Zn phase. In contrast, the lattice parameters of the secondary ε-(Ag,Cu)Zn phase were not affected by any treatment. As discussed above, inhomogeneity may play a role. In addition, due to the low melting point, the homologous temperature is at 0.43 Tm. Consequently, high temperature deformation systems are already active in Zn at room temperature. Cold rolling of hexagonal metals may give rise to the formation of twins, i.e., a low stacking fault energy, under lattice expansion in the c-axis and slip system activation. Subsequent annealing led to a recovery and homogenization of the microstructure. Lastly, another extension of the c-axis was detected in the overaged microstructure.

## 4. Discussion

### 4.1. Biodegradable Zn Alloys

Biocompatibility and safety of alloys for biodegradable implants are the key aspects of the development process. During the development of similar Zn alloys, the in vitro cytotoxicity, biocorrosion, and antimicrobial effect have already been investigated in depth [9,16,17,19,32,40,41,42,43]. Cytotoxicity tests of Zn-alloys, relying on standardized in vitro testing routines (ISO 10993-5 and 10993-12) typically used for non-degradable alloys, indicated a detrimental effect on cell viability. In vitro cytotoxicity is basically determined by the release of Zn-ions: i.e., the medium, the corrosion rate, potential formation of insoluble precipitates and the extract concentration. Besides, cell species and culture conditions need to be considered. In comparison to Zn, alloying elements tend to play a minor role given their low concentration [17,41,44,45]. The observed heterogeneity in the implementation of the cytotoxicity evaluation calls for strong efforts towards more standardized tests [41,44,46]. However, no single in vitro test can truly mimic the highly complex physiologic environments, such as blood vessels or bone. Each environment is characterized by specific fluids (pH, salts, proteins, molecules), cells (blood cells, endothelial cells, fibroblast, smooth muscle cells, bone cells), inflammatory response, or local rheological conditions. Consequently, it is very challenging to predict the in vivo performance of an alloy. Thus, the evaluation of the performance and the safety of an implant material still relies on animal testing. On the other hand, animal testing implies a further increase in complexity requiring a highly standardized study design and practice as well [2,47].

Material scientists in the field of biodegradable metals face similar problems. From an engineering perspective, the alloying and the microstructural refinement strategy depend on the targeted performance and required safety, i.e., mechanical properties and structural stability, corrosion rate, and biocompatibility. Accordingly, countless biodegradable alloys and thermomechanical treatment strategies have been developed resulting in a plethora of hardly comparable microstructural states. Thus, a balance between innovation and standardization must be found.

Given that these Zn alloys contain about 96% Zn, alloying elements tend to play a minor role concerning cytotoxicity [17,32]. Therefore, we focused in this study on the microstructural evolution by TMT and subsequent aging.

### 4.2. Alloy Design and Phases

Ag and Cu were selected as the main alloying elements due to their beneficial properties: (I) precipitation hardening [17,18,21,32], (II) higher electrode potentials leading to microgalvanic corrosion [14,17,42,48], (III) higher melting points mitigating the aging effect [32], (IV) good biocompatibility [17,19,32,49,50], (V) putative suppression of the inflammatory response by Cu [50], (VI) antimicrobial activity of Zn alloys [17,19,42]. Besides, the corrosion rate may be adjusted by a surface treatment [51].

The XRD-Rietveld analysis suggested that CR induced a c-parameter variation in the Zn-phase of all alloys (Table 2) which could theoretically be due to local variations of composition in the solid solution, or due to crystal defects such as stacking faults. The concentration of each alloying element combined with its radius (Cu < Zn < Ag) determines the degree to which it affects the length of the lattice parameters. CR-induced dynamic precipitation presumably causes a local matrix depletion of Ag and Cu, i.e., a certain inhomogeneity in the Zn matrix. Furthermore, a strong deviation of the c/a ratios of the Zn-phases from the ideal ratio of 1.63 for hcp elements was found. In contrast, the c/a ratio of the ε-phase was much closer to the ideal value indicating a denser packing. The EDX and the XRD analyses suggested, considering the sensitivity limits, that the stoichiometry of the ε-phase in the ternary alloys was approximately (Ag,Cu)Zn_4_, which is supported by other studies [32]. Regarding the quaternary alloys, Mn could be detected within the second phase by EDX, but no distinct phase could be detected by XRD. Even though a Li-related change in microstructure and hardness was observed, no Li phase could be detected by XRD and STEM-EELS so far.

### 4.3. Processing—Microstructure—Hardness Relationship

**Figure 11:**
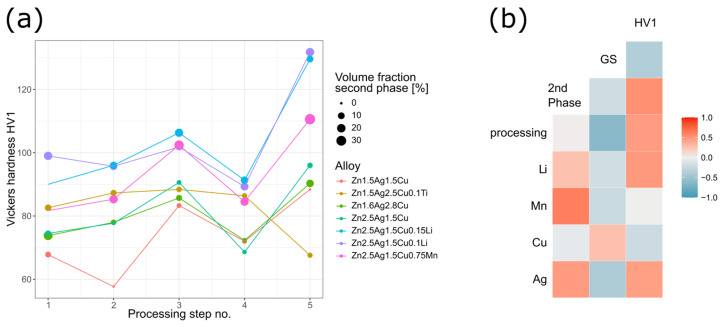
(**a**) Evolution of mechanical properties (Vickers hardness) and volume fraction of the second phase of the main alloys along the TMT route. (**b**) Heatmap reflecting Pearson correlation between features (Table A2). High correlation indicated by red coloring, high inverse correlation blue.

Figure 11 visualizes the results complied in Table A2 for the main alloy compositions. The effect of each processing step on the volume fraction of the ε-phase and on the hardness is plotted in Figure 11a. Hardness (and grain size) values were linked to the annealing-deformation cycles. For the sake of completeness, Zn-2.5Ag-1.5Cu-0.1Ti was included even though its properties differed from all other alloys. The different casting conditions of Zn-2.5Ag-1.5Cu-0.1Ti (see Materials and Methods) proved to have a major impact on the microstructure that could not be eliminated by the processing routine employed in this study (Figure 4 and Figure 5a).

In agreement with Chen et al. [32], the microstructure of AC Zn-1.5Ag-1.5Cu was characterized by a dendritic second phase embedded in the primary Zn phase. Solution annealing (processing step #2: 400 °C, 8 h) resulted in a complete dissolution of the secondary phase. The investigation of other Zn-Ag-Cu alloy variants demonstrated that the ε-phase could not be dissolved completely at higher contents of Ag and Cu.

In general, cold working gives rise to defects and dislocations, respectively. Distributed incoherent phases represent barriers, which impede dislocation movement and thus increase the hardness. Moreover, subjecting solution annealed and quenched, i.e., non-equilibrium, Zn alloys to plastic deformation (HR, CR) gives rise to dynamic precipitation [21]. Accordingly, a high number density of ε-(Ag,Cu)Zn precipitates was observed throughout the microstructure of CR alloys (Figure 6c). Alloying with Li resulted in exceptionally fine precipitates, while alloying with Mn affected the matrix grain size. After the second annealing (processing step #4) of the Zn-2.5Ag-1.5Cu-(0.3–0.75)Mn variants, a marked reduction in grain size was observed possibly due to the Mn related solute dragging effect on grain boundaries [21,52]. Vickers hardness of the CR ternary alloys ranged between 88–96 HV1 and the hardness of the quaternary alloys ranged between 95–132 HV1, surpassing the hardness of binary Zn-alloys subjected to a similar TMT procedure. A direct hardness comparison of Zn-2.5Ag-1.5Cu-0.1Li and Zn-2.5Ag-1.5Cu-0.75Mn, as both contain 0.9 at% of Li and Mn, respectively, highlights the strong effect of Li. However, the Li-containing phase and its distribution remain to be identified by advanced electron microscopic techniques such as STEM-EELS. In binary Zn-Li alloys, the hardness strongly correlates with the Li content but the underlying strength-ductility trade-off leads to gradual embrittlement [27,53].

Figure 11b shows the Pearson correlation between the features of all alloys (Table A2):

- Similar to Hall–Petch strengthening where strength and grain size are inversely correlated, smaller matrix grains (GS) resulted in a higher Vickers hardness (HV1).
- The solubility of the alloying elements in Zn can be derived from phase diagrams: Ag > Cu > Mn ≈ Li, which tends to be inversely correlated with the volume fraction of the corresponding secondary phase: Mn > Li > Cu > Ag [22]. Especially Mn led to a pronounced increase in the volume fraction of the second phase.
- Even though processing disrupted the dendrites and affected the morphology of the secondary phase, it had only a negligible impact on the volume fraction. Processing led to a refined microstructure and to an increase in hardness.
- Solid solution strengthening certainly plays a role, but the quantification is beyond the scope of this study.
- During solidification, the Ag-Cu enriched, higher melting secondary phase acts as a barrier limiting the diffusion and thus affecting the grain formation of the matrix. Accordingly, a high-volume fraction of incoherent secondary phase particles and smaller matrix grains result in increased hardness.
- Alloying with Li and Mn, respectively, resulted in an increase in the volume fraction of the ε-phase and smaller matrix grain, both contributing to a higher hardness.
- While Ag showed the expected behavior, apparently opposite correlations were observed for Cu (Figure 11b). Similarly, other groups found opposing effects for Ag and Cu [18,27].

### 4.4. Aging

Precipitation hardening is another well-established strategy to improve the mechanical properties. Here, however, hardness could not be increased further by controlled aging after CR as the solid solution matrix was already depleted of alloying elements due to dynamic precipitation.

The low activation energy of Zn suggests that diffusion and transition of point defects and dislocations are already eminent at room temperature. Recrystallization driven by the accumulated energy in deformed Zn alloys leads to a reduction of dislocation density. Hence, progressing recrystallization, recovery, and coarsening effects lead to deteriorating mechanical properties. Accordingly, artificial aging of CR alloys at 100 °C for only 1 min already led to a notable decrease in hardness (Figure 8a). Annealing of CR Zn-2.5Ag-1.5Cu-0.15Li prior to aging caused a hardness drop from 129 HV1 to 94 HV1. Subsequent natural and artificial aging (100 °C) resulted in a time- and temperature-dependent increase in hardness until overaging effects became dominant and the hardness decreased again. The artificially overaged microstructure was characterized by an intra-grain network of ε-phase needles (Figure 9b). Such a morphology is basically due to an orientation relationship between the matrix and the precipitate phase giving rise to preferential crystal growth directions. The closest packed direction tends to grow the fastest.

Some studies tend to highlight the obtained mechanical properties in comparison to the required implant benchmarks [25]. While this is certainly an important aspect, it seems to be insufficient as the assessment of the mechanical properties of deformed, non-equilibrium alloys represents rather a snapshot of the current state. Thus, the evaluation of the obtained mechanical properties should include a consideration of the structural-mechanical stability. Besides, corrosion properties are affected by the stability of the microstructure as well. Thus, the trilemma formulated for biodegradable orthopedic implants [54], sufficient mechanical performance—good biocompatibility—proper degradation rate may be extended by the aspect of stability.

## 5. Conclusions

In the present study, biodegradable alloy variants in the system Zn-Ag-Cu were cast and subjected to microstructure refinement. The following conclusions can be drawn:

1. Potential alloying elements were selected based on a comprehensive literature review. Compositions of promising alloys were optimized relying on thermodynamic simulations.
2. Plastic deformation by rolling caused dynamic precipitation from solid solution.
3. The Rietveld analysis indicated that cold rolling induced variations in the crystallographic c-axis of the Zn phase. This may be caused by precipitation-related heterogeneity in the matrix or by the formation of stacking faults. A subsequent heat treatment obliterated that effect.
4. Due to the low solubility and the ε-phase stabilizing effect, alloying with Mn resulted in an increase in volume fraction and a matrix grain refinement. Consequently, hardness increased. According to the EDX analysis, Mn was dissolved in the secondary phase, but no distinct phase could be identified by XRD analysis.
5. Alloying with Li resulted in a slight increase in the volume fraction of the second phase. However, a noticeable change in the morphology of the matrix microstructure was observed. Furthermore, alloying with Li led to exceptionally fine precipitates. Li could be detected by ICP-OES but not by any structural analysis method employed in this investigation, such as XRD and EELS, so far.
6. Quaternary alloys showed improved properties compared to ternary alloys. A comparison of the atomic concentrations revealed that Li had a stronger impact on hardness than Mn.
7. Meeting the required mechanical benchmarks after TMT is a crucial step in the biodegradable Zn alloy development of, but this is not sufficient. Scientists face a multilemma that remains to be solved.
8. Nowadays the ability to make a comparative assessment of such biodegradable alloys is rather limited due to the various compositions and TMTs. Therefore, it may be advisable to establish more standardized tests to evaluate the tradeoffs between performance, stability, corrosion, and cytotoxicity.

## Figures and Tables

**Table 1:** Comparison of ThermoCalc simulations and DTA results.

Alloy	ThermoCalc TCMG6		DTA Measurement			
	T_sol_ [°C]	Tliq [°C]	T_onset_ ↑ =T_sol_	T_peak_ ↑ =T_liq_	T_onset1_ ↓ =T_liq supercooled_	T_onset2_ ↓ =T_peritectic_
Zn-2.5Ag-1.5Cu	426	431	421	444	429	405
Zn-2.5Ag-1.5Cu-0.1Li	397	427	414	438	422	403
Zn-2.5Ag-1.5Cu-0.75Mn	419	460	420	444	435	400

**Table 2:** XRD of CR alloys. Lattice parameters of the phases according to the Rietveld analysis.

Phase	Sample	a [Å]	c [Å]
Zinc	ICDD-PDF No 65-3358 P6_3_/mmc	2.66(5)	4.94(7)
	Zn-1.5Ag-1.5Cu	2.67(9)	4.92(9)
	Zn-1.5Ag-2.5Cu	2.68(0)	4.89(3)
	Zn-2.5Ag-1.5Cu	2.68(2)	4.92(8)
	Zn-2.5Ag-1.5Cu-0.75Mn	2.68(5)	4.92(9)
	Zn-2.5Ag-1.5Cu-0.1Li	2.68(3)	4.91(9)
	Zn-2.5Ag-1.5Cu-0.15Li	2.67(6)	4.92(7)
Zinc II	Zn-1.5Ag-1.5Cu	2.66(8)	4.84(9)
	Zn-1.5Ag-2.5Cu	2.66(8)	4.83(6)
	Zn-2.5Ag-1.5Cu	2.66(8)	4.83(9)
	Zn-2.5Ag-1.5Cu-0.75Mn	2.66(8)	4.83(4)
	Zn-2.5Ag-1.5Cu-0.1Li	2.67(0)	4.83(5)
	Zn-2.5Ag-1.5Cu-0.15Li	2.66(8)	4.87(0)
ε-phase	ICDD-PDF No 51-0642	2.78(1)	4.35(3)
	Zn-1.5Ag-1.5Cu	2.78(5)	4.33(2)
	Zn-1.5Ag-2.5Cu	2.77(9)	4.32(0)
	Zn-2.5Ag-1.5Cu	2.79(1)	4.34(1)
	Zn-2.5Ag-1.5Cu-0.75Mn	2.79(4)	4.34(5)
	Zn-2.5Ag-1.5Cu-0.1Li	2.79(3)	4.34(9)
	Zn-2.5Ag-1.5Cu-0.15Li	2.79(1)	4.35(0)

**Table 3:** The effect of TMT and aging on the microstructure of Zn-2.5Ag-1.5Cu-0.15Li was investigated by XRD measurements and assessment of hardness. Two phases, Zn and ε-(Ag,Cu)Zn, were detected by XRD. While TMT and aging affected the length of the c-axis of the Zn-phase, the lattice parameters of ε-(Ag,Cu)Zn proved to be unaffected. Further evidence regarding the state of the microstructure is given by the hardness (HV1). Hardness values falling again (after the maximum) are an indication of overaging (+). Hardness values before aging are given in brackets (day 0: starting point).

Zn-2.5Ag-1.5Cu-0.15Li TMT Step|Heat Treatment|Aging	Zn ICSD-247147-P63 mmc		ε-(Ag,Cu)Zn ICSD-103157-P63 mmc		HV1 (Day 0)
	a [Å]	c [Å]	a [Å]	c [Å]	
#1 (AC)|–|398 days, 22 °C	2.68	4.85	2.79	4.37	102 (90)
#2 (HT)|–|397 days, 22 °C	2.68	4.84	2.78	4.39	111 (96)
#5 (CR)|–|0 days	Table 2	Table 2	2.79	4.35	130
#6 (CR)|0.5 h, 400 °C|0 days	2.69	4.82	2.78	4.38	94
#6 (CR)|0.5 h, 400 °C|322 days, 22 °C	2.67	4.92	2.79	4.37	105^+^
#6 (CR)|0.5 h, 400 °C|83 days, 100 °C	2.68	4.88	2.79	4.38	93^+^

## Data Availability

The raw/processed data required to reproduce these findings cannot be shared at this time as the data also forms part of an ongoing study.

## Appendix A

**Table A1:** Quantitative analysis of all compositions of alloys (ICP-OES) and their nominal composition.

Quantitative Analysis of Alloys [wt%]	Nominal Composition
Zn-1.5Ag-1.5Cu (ICP)	Zn-1.5Ag-1.5Cu
Zn-1.6Ag-2.8Cu (ICP)	Zn-1.5Ag-2.5Cu
Zn-1.4Ag-2.5Cu-0.13Ti (XRF)	Zn-1.5Ag-2.5Cu-0.1Ti
Zn-2.5Ag-1.5Cu (ICP)	Zn-2.5Ag-1.5Cu
Zn-2.5Ag-1.5Cu-0.3Mn (ICP)	Zn-2.5Ag-1.5Cu-0.3Mn
Zn-2.5Ag-1.5Cu-0.75Mn (ICP)	Zn-2.5Ag-1.5Cu-0.75Mn
Zn-2.5Ag-1.5Cu-0.06Li (ICP)	Zn-2.5Ag-1.5Cu-0.06Li
Zn-2.4Ag-1.5Cu-0.1Li (ICP)	Zn-2.5Ag-1.5Cu-0.1Li
Zn-2.5Ag-1.5Cu-0.15Li (ICP)	Zn-2.5Ag-1.5Cu-0.15Li

**Table A2:** Structural and mechanical data of all processed alloys. A detailed description of data extraction procedures and hardness measurement, respectively, is given in the method section. NA: no data available, * grains barely visible, ** large grains, less than 20 grains analyzed, *** no SEM images available, + precipitates detected by SEM, - no precipitates detected by SEM.

Alloy	Grain Size [µm]	Sec. Phase [%]	Nano-Sized Precipitates	HV1
Processing step #1: As Cast				
Zn-1.5Ag-1.5Cu	195	6	-	68
Zn-1.5Ag-2.5Cu	272	23	-	74
Zn-1.4Ag-2.5Cu-0.1Ti	216	9	NA ***	84
Zn-2.5Ag-1.5Cu	167	NA	NA ***	72
Zn-2.5Ag-1.5Cu-0.3Mn	290 *	NA	NA ***	74
Zn-2.5Ag-1.5Cu-0.75Mn	118 *	NA	NA ***	82
Zn-2.5Ag-1.5Cu-0.06Li	164 *	NA	NA ***	85
Zn-2.5Ag-1.5Cu-0.1Li	189 *	19	NA ***	99
Zn-2.5Ag-1.5Cu-0.15Li	NA *	NA	NA ***	90
Processing step #2: Homogenization (400 °C, 8 h)				
Zn-1.5Ag-1.5Cu	267 **	0	NA ***	58
Zn-1.5Ag-2.5Cu	298 **	5	-	78
Zn-1.4Ag-2.5Cu-0.1Ti	211 **	7	NA ***	91
Zn-2.5Ag-1.5Cu	271 **	1	-	77
Zn-2.5Ag-1.5Cu-0.3Mn	310 **	NA	NA ***	73
Zn-2.5Ag-1.5Cu-0.75Mn	88	19	NA ***	85
Zn-2.5Ag-1.5Cu-0.06Li	129 **	NA	NA ***	92
Zn-2.5Ag-1.5Cu-0.1Li	152 **	15	NA ***	96
Zn-2.5Ag-1.5Cu-0.15Li	149 **	13	NA ***	96
Processing step #3: Hot Rolling (200 °C, 50%)				
Zn-1.5Ag-1.5Cu	NA *	3	+	84
Zn-1.5Ag-2.5Cu	NA *	7	+	86
Zn-1.4Ag-2.5Cu-0.1Ti	NA *	5	NA ***	86
Zn-2.5Ag-1.5Cu	NA *	4	+	91
Zn-2.5Ag-1.5Cu-0.3Mn	NA *	NA	NA ***	88
Zn-2.5Ag-1.5Cu-0.75Mn	NA *	27	NA ***	103
Zn-2.5Ag-1.5Cu-0.06Li	NA *	NA	NA ***	108
Zn-2.5Ag-1.5Cu-0.1Li	NA *	10	NA ***	102
Zn-2.5Ag-1.5Cu-0.15Li	NA *	18	NA ***	106
Processing step #4: Annealing (400 °C, 0.5 h)				
Zn-1.5Ag-1.5Cu	189	3	NA ***	72
Zn-1.5Ag-2.5Cu	74	3	-	72
Zn-1.4Ag-2.5Cu-0.1Ti	42	4	NA ***	87
Zn-2.5Ag-1.5Cu	75	2	-	69
Zn-2.5Ag-1.5Cu-0.3Mn	90	NA	NA ***	74
Zn-2.5Ag-1.5Cu-0.75Mn	35	18	NA ***	85
Zn-2.5Ag-1.5Cu-0.06Li	79	NA	NA ***	88
Zn-2.5Ag-1.5Cu-0.1Li	91	17	NA ***	89
Zn-2.5Ag-1.5Cu-0.15Li	99	14	NA ***	91
Processing step #5: Cold Rolling (22 °C, 50%)				
Zn-1.5Ag-1.5Cu	NA *	0	NA ***	88
Zn-1.5Ag-2.5Cu	NA *	14	+	90
Zn-1.4Ag-2.5Cu-0.1Ti	NA *	4	NA ***	69
Zn-2.5Ag-1.5Cu	NA *	5	+	96
Zn-2.5Ag-1.5Cu-0.3Mn	NA *	NA	NA ***	95
Zn-2.5Ag-1.5Cu-0.75Mn	NA *	34	+	111
Zn-2.5Ag-1.5Cu-0.06Li	NA *	NA	NA ***	124
Zn-2.5Ag-1.5Cu-0.1Li	NA *	19	+	132
Zn-2.5Ag-1.5Cu-0.15Li	NA *	10	+	129
